# Alcohol and Female Sexuality: A Look at Expectancies and Risks

**Published:** 1994

**Authors:** Jeanette Norris

**Affiliations:** Jeanette Norris, Ph.D., is a research scientist at the Alcohol and Drug Abuse Institute of the University of Washington, Seattle, Washington

## Abstract

Despite public perceptions that alcohol consumption enhances sexual experiences and indicates sexual permissiveness, there is no simple correlation between alcohol consumption and sexual behavior in women. It has been shown, however, that alcohol negatively affects female sexuality, leading to sexual dysfunction and sexual victimization of women.

Studies of associations between alcohol use and female sexuality have been far less abundant than similar studies in men (for general reviews, see Carpenter and Armenti 1972; Crowe and George 1989; Wilsnack 1984; and Wilson 1981). However, the importance of examining the relationship between women’s alcohol consumption and various aspects of their sexuality has become increasingly apparent. Researchers now recognize that findings about male sexuality and drinking do not necessarily apply to women. For instance, laboratory studies have shown that women’s and men’s sexual responses to the knowledge that they have consumed alcohol differ dramatically (for reviews, see Crowe and George 1989, and Leigh 1990*a*).

Even similar alcohol-related problems in men and women may have different underlying reasons or lead to gender-specific consequences. For example, sexual dysfunction (e.g., men’s erectile failure or women’s lack of orgasm) may not have the same meaning for men and women and may affect subsequent drinking behavior differently. Thus, treatment of women and men with alcohol-related sexual dysfunction probably requires gender-specific approaches.

This article addresses two important issues of the relationship between alcohol consumption and female sexuality. First, it analyzes alcohol’s effects on women’s sexual behavior and reviews both experimental studies of alcohol’s effect on sexual arousal and studies of alcohol use in conjunction with sex in everyday life. Second, it examines how alcohol consumption serves as a risk factor for sexual dysfunction, risky sexual behavior, and sexual victimization.

## Effects of Alcohol on Sexual Arousal

Few laboratory studies have investigated alcohol’s pharmacological and psychological effects on women’s sexual response. Analysis of the pharmacological effects showed that, similar to men, women experience decreased sexual responsiveness even at moderate^1^ levels of alcohol consumption. In fact, increasing blood alcohol levels appear to cause a decrease in vaginal blood flow or orgasmic intensity and an increase in orgasmic latency (Crowe and George 1989; Leigh 1990*a*; Wilsnack 1984; Wilson 1981).

Women’s subjective experience of sexual arousal, however, does not parallel their physiological response. As blood alcohol levels increase and physiological indicators of arousal show lowered responsiveness, women report being more sexually aroused (Crowe and George 1989; Leigh 1990*a*; Wilsnack 1984; Wilson 1981). Men’s self-reported sexual arousal, in contrast, corresponds to the physiological measures.

Psychological effects of alcohol are manifested in the form of outcome expectancies associated with alcohol consumption. Expectancies are individuals’ beliefs about the effects of alcohol on their own or others’ behavior. Research has shown that women and men hold essentially the same outcome expectancies about alcohol’s effects on sexual behavior (for review, see Crowe and George 1989). In these studies, approximately 50 percent of the women and men surveyed believe that alcohol enhances or disinhibits sexual behavior.

Laboratory experiments have studied the effects of expectancies with the balanced placebo design. This experimental design manipulates two factors: the belief instilled by the experimenter that alcohol has or has not been consumed (“expectancy set”) and the actual presence or absence of alcohol in the subject’s beverage. Thus, four experimental groups are created: subjects who expect and receive alcohol, subjects who expect alcohol but do not receive it, subjects who do not expect alcohol but do receive it, and subjects who do not expect alcohol and do not receive it. This design allows researchers to identify alcohol effects that are solely the result of the expectancy set and that occur presumably because of the individual’s underlying outcome expectancies.

Balanced placebo studies have shown different influences of expectancy set for men and women. In men, the expectation of receiving alcohol strongly affects sexual arousal. Men who believe they have consumed an alcoholic beverage, whether or not they actually have, generally exhibit greater sexual arousal in response to sexually explicit materials than do men who believe they have consumed a nonalcoholic drink. In women, no such effects of expectancy set have been found (Crowe and George 1989; Leigh 1990*a*; Wilsnack 1984; Wilson 1981).

Crowe and George (1989) have attempted to explain the lack of expectancy set effects in women. They argue that because of societal restrictions on displaying sexual pleasure and because of the risk of unwanted pregnancy, women are more conflicted and inhibited about their sexuality than men. Consequently, women are less likely than men to succumb to the expectancies about alcohol’s disinhibiting effects on sexuality, even though they generally hold the same beliefs about alcohol’s effects as men. The influence of expectancy set is not sufficient to overcome these inhibitions, and the low alcohol doses used in balanced placebo studies do not have a direct pharmacological disinhibitory effect. At higher alcohol doses, however, women’s psychological sexual responses may increase because of alcohol-induced reduction of the inhibitory conflict (Steele and Josephs 1990; Steele and Southwick 1985). This interpretation is appealing, because it is based on both theory and empirical evidence, but it has yet to be subjected to extensive and rigorous testing.

Yet conclusions about alcohol’s effects on women’s sexual responses suffer from several drawbacks. Only a few studies employing female subjects have been conducted until now; consequently, their conclusions are based on the evaluation of a relatively small number of women. Also, these women probably do not represent the general population, because most of them were young and rather liberal in their sexual attitudes (Catania et al. 1990).

It also is unclear to what extent the results of laboratory studies that have focused mainly on sexual arousal after sexually explicit stimuli reflect sexual behavior in the general population and in everyday life. The next section addresses issues concerning the use of alcohol and sexual behavior beyond the walls of the laboratory.

## Drinking and Sexual Behavior in the “Real World”

Several questions arise when analyzing the association between drinking and sexual behavior. Does alcohol consumption generally increase the likelihood that sex will occur? Are specific situations likely to favor co-occurrence of drinking and sex? How do expectancies, especially the belief that alcohol enhances sexual experience, affect the use of alcohol in conjunction with sex?

Virtually every researcher on this topic mentions assumptions people make about drinking and sexual behavior: that alcohol disinhibits sexual feelings; that drinking generates the expectation that sex will occur; and that, in general, alcohol consumption indicates sexual permissiveness (for discussion, see Leigh 1990*a*,*b*). However, data from population surveys do not support the validity of all these assumptions.

### Alcohol Consumption and Frequency of Sexual Activity

Although several studies have found a positive correlation between general alcohol consumption patterns and the overall frequency of sexual activity (for review, see Leigh 1990*a*), this relationship does not necessarily mean that drinking and sex occur together on a specific occasion (Harvey and Beckman 1986; Temple and Leigh 1992). A study by Leigh (1993) showed that the frequency of sexual activity decreased in heterosexual women and men when combined with alcohol consumption. (For homosexual women and men, sexual activity decreased only slightly or not at all after alcohol consumption.)

Other studies, however, have detected a positive association between drinking and sexual activity. Leigh and Schafer (1993) found that for both women and men, the likelihood of sex occurring on a given occasion was directly dependent on the amount of alcohol consumed. Flanigan and Hitch (1986) came to a similar conclusion when they analyzed occasions leading to young women’s first time intercourse.

Thus, it seems that there is no simple correlation between alcohol consumption and sexual behavior. Many of the differences in the results of these studies may be caused by the varying populations and measures employed. The influence of additional factors, such as drinking habits and expectancies, also may help to explain apparent discrepancies. Characteristics of specific situations leading to alcohol consumption and sex also may influence individual drinking and sexual behavior.

### Alcohol Consumption and Sexual Activity on Specific Occasions

The combination of heavy drinking and consequent sexual activity appears particularly relevant in situations involving a new partner. Temple and Leigh (1992) found that both men and women were more likely to drink during sex with a new partner than with a recent partner. Sexual encounters also were more likely to be unexpected if alcohol was involved. In a similar vein, Leigh and Schafer (1993) reported that sex with a new partner was more likely after heavy drinking (eight or more drinks) on a particular occasion than after lighter drinking (fewer than eight drinks). This trend was more apparent in men than in women, perhaps because women generally drank less than men.

Alcohol consumption in women also appears to be associated either with non-traditional sexual activities or with strong inhibitions about sexual activities. In a study by Covington and Kohen (1984), alcoholic women used more alcohol with sex and participated in a greater variety of sexual activities than did nonalcoholic women. For example, alcoholic respondents reported greater frequency of masturbation when drinking than did nonalcoholic women. In another study, alcoholic women reported more often than nonalcoholic women that they desired sex, had sex, and enjoyed sex when they were drinking yet indicated less sexual satisfaction in general (Beckman 1979).

Klassen and Wilsnack (1986) reported a more complex relationship between heavy drinking in women and their sexual behavior. In this study,^2^ moderate and heavy drinkers generally were more likely than light drinkers and abstainers to engage in nontraditional sexual activity. Extreme heavy drinkers, however, were the sexually most traditional and indicated conflicts and inhibitions about sex.

This evidence suggests that if alcohol is associated with the occurrence of sex, it is most likely under conditions of heavy drinking on a particular occasion or in habitual heavy drinkers. In these situations, alcohol may be used to alleviate sexual conflicts (Klassen and Wilsnack 1986), an assumption that may be based on people’s outcome expectancies about alcohol and sexual activity.

### Outcome Expectancies and Sexual Activity

Laboratory studies and population surveys have shown that many women have positive expectations about alcohol’s effects on sexual behavior, such as enhanced sexual enjoyment and decreased nervousness (for reviews, see Leigh 1990*a*, and Wilsnack 1984). Recent studies have begun to clarify the relationship between women’s drinking patterns, outcome expectancies, and sexual activities.

Women drinkers’ expectancies about alcohol’s positive and disinhibiting effects on sexuality seem to increase through drinking categories (Klassen and Wilsnack 1986). Leigh (1990*b*) also reported that compared with lighter drinkers, both male and female heavier drinkers more strongly endorsed three types of expectancies concerning alcohol’s effects on sexual behavior: sexual enhancement (i.e., the expectation that alcohol will make sex “better”), decreased nervousness, and increased risk taking. Alcoholic women also had more positive associations with the use of alcohol during sex than did nonalcoholics (Covington and Kohen 1984). However, women’s expectancies generally did not predict their overall proportion of sexual encounters involving alcohol (Leigh 1990*b*).

The highest level of drinking occurred among women who had strong expectancies and negative attitudes about sex—who felt nervous or guilty. Thus, it appears that expectancies about the effects of alcohol on sex may motivate heavy drinking in some women, particularly those most conflicted about sex (Klassen and Wilsnack 1986; Leigh 1990*b*). These women may use alcohol to reduce their sexual inhibitions (Steele and Josephs 1990; Steele and Southwick 1985). This interpretation is supported by findings that individuals often use alcohol when having sex either for the first time (Flanigan and Hitch 1986) or for the first time with a new partner (Temple and Leigh 1992), two occasions that are likely to create feelings of anxiety or conflict.

## Drinking and Sexual Dysfunction

In contrast to the positive expectancies associated with alcohol use, women often experience serious repercussions from drinking. One consequence of heavy drinking, and especially alcohol dependence, can be sexual dysfunction (for review, see Wilsnack 1984). This includes a wide array of psychologically and physiologically based problems, such as lack of sexual interest, decreased sexual arousal, and inability to achieve orgasm.

Klassen and Wilsnack (1986) reported the highest levels of sexual dysfunction among the heaviest drinkers in their study. In another analysis, alcoholic women also reported substantially higher rates of sexual dysfunction than nonalcoholic women (Covington and Kohen 1984).

Most of the alcoholic women with sexual dysfunctions reported having these problems already before the onset of their alcoholism (Covington and Kohen 1984). This finding indicates that a preexisting sexual dysfunction may motivate a woman to drink, perhaps because of expectations that alcohol will enhance her sexual experience. Self-medicative drinking because of sexual dysfunction may in turn cause subsequent problem drinking and alcohol dependence (Wilsnack 1984). In fact, sexual dysfunction was the strongest predictor of chronic problem drinking in a recent longitudinal study (Wilsnack et al. 1991). Yet because of alcohol’s deleterious effects on sexual functioning (Wilson 1981), the women may have become disappointed with their sexual performance and may have consumed even more alcohol to alleviate feelings of inadequacy or in a continuing hope of improving their sexuality. As Wilsnack and colleagues (1991) state, “. . . a self-reinforcing cycle may occur in which heavy drinking becomes both cause and consequence of sexual dysfunction” (p. 315).

To prevent sexual problems from escalating into drinking problems, new intervention modalities may have to be developed and tested. Goldman and Roehrich (1991) have presented data that show promise for changing individuals’ expectancies and subsequently their consumption levels. This approach involves challenging individuals’ expectancies about alcohol effects by demonstrating that it is the belief and not necessarily alcohol itself that has an effect. However, this research is in an early stage and has not yet shown long-term effects.

## Drinking and Risky Sex

Increasing the risk of sexual dysfunction and problem drinking is not the only serious repercussion of combining alcohol and sex. Another concern that has become more and more relevant in recent years is whether alcohol use during sex may increase the risk of contracting sexually transmitted diseases, including the human immunodeficiency virus (HIV) that causes acquired immune deficiency syndrome (AIDS) (for a review, see Leigh and Stall 1993). Because the correct use of a condom during sex is considered the most effective way of preventing transmission of HIV, risky sex for a woman is defined as sexual intercourse without the use of a condom. Alcohol is considered a risk factor for contracting HIV because of its assumed disinhibitory effects, which may cause individuals not to use good judgment in their sexual behavior and thus to have risky sex.

Only a few studies so far have addressed the question of whether alcohol consumption decreases condom use. Leigh (1990*c*) found a correlation for heterosexual men and women between alcohol use during sex and risky sex. But because the overwhelming proportion of persons in the study were practicing unsafe sex with and without alcohol consumption, this result cannot be considered statistically significant.

A study among pregnant substance-abusing adolescents also found a positive relationship between alcohol use during sex and risky sexual behavior (Gillmore et al. 1992). Although this correlation was significant, it also was influenced by additional factors, such as delinquency, family closeness, and alcohol and other drug use by partners.

Two studies revealed no association of alcohol use with risky sex. In a study of heterosexual women, alcohol consumption during the most recent sexual encounter with a new partner did not affect their use of a condom (Temple and Leigh 1992). Drinking and risky sex also were not correlated in heterosexual men or women that were studied using a diary technique in which subjects kept track of their drinking and sexual activities (Leigh 1993).

From these studies, no firm conclusions can yet be drawn about the effects of alcohol consumption on risky sexual behavior. Because unprotected sex, especially with a new partner, can put an individual at particularly high risk of contracting HIV, more studies are needed that rigorously address the association between alcohol and condom use. Specific questions include whether alcohol reduces the frequency or the reliability of condom use.

More attention also must be paid to the influence of individual differences and situational variables on risky sexual behavior (Gillmore et al. 1992). These variables may differ somewhat for women and men; for example, whether a condom is used may depend on the balance of power between the partners in a relationship. Prevention strategies to reduce risky sexual behavior and thus protect women’s health should be sensitive to issues such as these.

## Alcohol as a Risk Factor for Sexual Victimization

Compared with a sober woman, a drinking woman is considered more sexually disinhibited and available by both men and women (George et al. 1986, 1988). This type of misperception can put a woman at risk for unwanted sexual advances. For instance, up to 50 percent of sexual assaults by acquaintances involve alcohol consumption by the victim or the assailant (Koss et al. 1987; Muehlenhard and Linton 1987).

Drinking by the victim also is more likely to result in completed rather than attempted rape. A possible reason is that when women are drinking at the time of the rape, they are not able to resist as well or be as clear about their nonconsent as when they are sober (Abbey and Ross 1992).

That alcohol can have direct pharmacological effects on individuals’ ability to make judgments about sexual assault was shown in a study analyzing the effects of alcohol consumption on interpretations of eroticized rape depictions (Norris and Kerr 1993). Women and men were asked to judge the behavior of both assailant and victim in the story. Female and male subjects showed significant changes in their perception of the situation after consumption of only a moderate amount of alcohol (i.e., less than half the amount required to be considered legally intoxicated). Compared with sober women, drinking women judged the assailant as having used less force and his behavior as more acceptable. They also indicated an increased willingness to behave like the female story character, that is, as a willing victim. Male drinkers viewed the female character as more deviant than male nondrinkers and expressed a greater willingness to behave like the assailant.

The differences in perception induced by alcohol might be the result of alcohol’s “focusing” effect, or “myopia” (e.g., Steele and Josephs 1990). This means that alcohol consumption limits subjects’ ability to focus on all but the most salient and/or disinhibitory cues in a given situation. In Norris and Kerr’s study, such cues would have been those related to obtaining pleasure rather than those related to pain or distress experienced by the victim.

Alcohol consumption by the victim or assailant also can change other persons’ perceptions of an assault situation. Norris and Cubbins (1992) found that drinking appears to imply permissiveness in a rape situation. In their experiment with depictions of an acquaintance rape, in which either the victim, her assailant, or both were drinking, women and men considered the incident least likely to be a rape if the victim and the assailant had been drinking together.

Similarly, alcohol consumption appears to produce different attitudes toward the assailant and the victim after a rape has occurred. Richardson and Campbell (1982) found that both women and men attributed more responsibility for the assault to an intoxicated rape victim than to a sober one. However, the offender was blamed less when intoxicated than when sober. In a similar study among male and female college students, an assailant was judged most positively if he had been drinking with the victim (Norris and Cubbins 1992). The victim’s reactions after the rape, however, were seen as least negative and the couple was considered most likely to date again within a week if the woman was portrayed as drinking with the assailant.

These studies show that alcohol can contribute to sexual victimization in several ways. Because socializing between women and men often involves alcohol consumption, understanding how sexual assault can result from this interaction is key to learning how to prevent future victimization.

## Conclusions and Future Research Directions

Research over the last 10 years has made some progress in understanding how alcohol consumption and sexual behavior of women are connected. It has become amply clear that the relationship between the two behaviors is determined by a complex set of both situational variables and individual characteristics. Future research must focus on these complexities.

Sexual problems can put women at high risk for drinking problems (Wilsnack 1984) and therefore warrant more attention in alcoholism research and treatment. On the other hand, alcohol consumption also can cause sexual dysfunction. To better understand the complex relationship between sexual dysfunction and alcohol consumption, it is important to study the role of outcome expectancies concerning alcohol’s effect on sex, especially among heavy drinkers.

Negative consequences of combining sexual activities with alcohol use also may include risky sexual behavior, which can lead to infection with sexually transmitted diseases, such as HIV/AIDS. A primary question appears to be if and under what circumstances alcohol consumption contributes to the nonuse or the unreliable use of a condom. Research also should address ways of increasing women’s assertiveness in negotiating condom use.

It is equally important to understand how alcohol contributes to an increased risk for sexual assault on women. Excessive drinking incapacitates a woman, making it difficult to respond effectively to an assault. But even a relatively low level of alcohol consumption can make her vulnerable by changing her perceptions of force. Further study is needed to understand exactly how alcohol influences perceptions of sexual assault.

## Footnotes

1 Moderate alcohol consumption typically means a blood alcohol content of 0.04. In women, this is reached after drinking about two to three standard drinks. A standard drink is defined as 12 fluid ounces of beer, 5 fluid ounces of wine, or 1.5 fluid ounces of distilled spirits.

2 In this study, light drinkers consumed less than 0.22 fluid ounce, moderate drinkers between 0.22 and 0.99 fluid ounce, heavy drinkers between 1.0 and 1.5 fluid ounces, and extreme heavy drinkers more than 1.5 fluid ounces of pure alcohol per day during the previous 30 days. A standard drink contains approximately 0.5 fluid ounce of pure alcohol.

## References

[b1-arhw-18-3-197] Abbey A, Ross LT The Role of Alcohol in Understanding Misperception and Sexual Assault.

[b2-arhw-18-3-197] Beckman LJ (1979). Reported effects of alcohol on the sexual feelings and behavior of women alcoholics and nonalcoholics. Journal of Studies on Alcohol.

[b3-arhw-18-3-197] Carpenter JA, Armenti NP, Kissen B, Begleiter H (1972). Some effects of ethanol on human sexual and aggressive behavior. The Biology of Alcoholism.

[b4-arhw-18-3-197] Catania JA, Gibson DR, Chitwood DD, Coates TJ (1990). Methodological problems in AIDS behavioral research: Influences on measurement error and participation bias in studies of sexual behavior. Psychological Bulletin.

[b5-arhw-18-3-197] Covington SS, Kohen J, Stimmel B (1984). Women, alcohol and sexuality. Cultural and Sociological Aspects of Alcoholism and Substance Abuse.

[b6-arhw-18-3-197] Crowe LC, George WH (1989). Alcohol and human sexuality: Review and integration. Psychological Bulletin.

[b7-arhw-18-3-197] Flanigan BJ, Hitch MA (1986). Alcohol use, sexual intercourse, and contraception: An exploratory study. Journal of Alcohol and Drug Education.

[b8-arhw-18-3-197] George WH, Skinner JB, Marlatt GA Male Perceptions of the Drinking Woman: Is Liquor Quicker?.

[b9-arhw-18-3-197] George WH, Gournic SJ, McAfee MP (1988). Perceptions of postdrinking female sexuality: Effects of gender, beverage choice, and drink payment. Journal of Applied Social Psychology.

[b10-arhw-18-3-197] Gillmore MR, Butler SS, Lohr MJ, Gilchrist L (1992). Substance use and other factors associated with risky sexual behavior among pregnant adolescents. Family Planning Perspectives.

[b11-arhw-18-3-197] Goldman MS, Roehrich L (1991). Alcohol expectancies and sexuality. Alcohol Health & Research World.

[b12-arhw-18-3-197] Harvey SM, Beckman LJ (1986). Alcohol consumption, female sexual behavior and contraceptive use. Journal of Studies on Alcohol.

[b13-arhw-18-3-197] Klassen AD, Wilsnack SC (1986). Sexual experience and drinking among women in a U.S. national survey. Archives of Sexual Behavior.

[b14-arhw-18-3-197] Koss MP, Gidycz CJ, Wisniewski N (1987). The scope of rape: Incidence and prevalence of sexual aggression and victimization. Journal of Consulting and Clinical Psychology.

[b15-arhw-18-3-197] Leigh BC (1990a). “Venus gets in my thinking”: Drinking and female sexuality in the age of AIDS. Journal of Substance Abuse.

[b16-arhw-18-3-197] Leigh BC (1990b). The relationship of sex-related alcohol expectancies to alcohol consumption and sexual behavior. British Journal of Addiction.

[b17-arhw-18-3-197] Leigh BC (1990c). The relationship of substance use during sex to high-risk sexual behavior. Journal of Sex Research.

[b18-arhw-18-3-197] Leigh BC (1993). Alcohol consumption and sexual activity as reported with a diary technique. Journal of Abnormal Psychology.

[b19-arhw-18-3-197] Leigh BC, Schafer JC (1993). Heavy drinking occasions and the occurrence of sexual activity. Psychology of Addictive Behaviors.

[b20-arhw-18-3-197] Leigh BC, Stall R (1993). Substance use and risky sexual behavior for exposure to HIV: Issues in methodology, interpretation, and prevention. American Psychologist.

[b21-arhw-18-3-197] Muehlenhard CL, Linton MA (1987). Date rape and sexual aggression in dating situations: Incidence and risk factors. Journal of Counseling Psychology.

[b22-arhw-18-3-197] Norris J, Cubbins LA (1992). Dating, drinking, and rape: Effects of victim’s and assailant’s alcohol consumption on judgments of their behavior and traits. Psychology of Women Quarterly.

[b23-arhw-18-3-197] Norris J, Kerr K (1993). Alcohol and violent pornography: Responses to permissive and nonpermissive cues. Journal of Studies on Alcohol.

[b24-arhw-18-3-197] Richardson D, Campbell JL (1982). Alcohol and rape: The effect of alcohol on attributions of blame for rape. Personality and Social Psychology Bulletin.

[b25-arhw-18-3-197] Steele CM, Josephs RA (1990). Alcohol myopia: Its prized and dangerous effects. American Psychologist.

[b26-arhw-18-3-197] Steele CM, Southwick L (1985). Alcohol and social behavior. I: The psychology of drunken excess. Journal of Personality and Social Psychology.

[b27-arhw-18-3-197] Temple MT, Leigh BC (1992). Alcohol consumption and unsafe sexual behavior in discrete events. Journal of Sex Research.

[b28-arhw-18-3-197] Wilsnack SC, Wilsnack SC, Beckman LJ (1984). Drinking, sexuality, and sexual dysfunction in women. Alcohol Problems in Women: Antecedents, Consequences and Intervention.

[b29-arhw-18-3-197] Wilsnack SC, Klassen AD, Schur BE, Wilsnack RW (1991). Predicting onset and chronicity of women’s problem drinking: A five-year longitudinal analysis. American Journal of Public Health.

[b30-arhw-18-3-197] Wilson GT, Mello NK (1981). The effects of alcohol on human sexual behavior. Advances in Substance Abuse: Behavioral and Biological Research.

